# Pro-oncogenic action of LOX-1 and its splice variant LOX-1Δ4 in breast cancer phenotypes

**DOI:** 10.1038/s41419-018-1279-1

**Published:** 2019-01-18

**Authors:** Sabina Pucci, Chiara Polidoro, Chiara Greggi, Francesca Amati, Elena Morini, Michela Murdocca, Michela Biancolella, Augusto Orlandi, Federica Sangiuolo, Giuseppe Novelli

**Affiliations:** 1 Department of Biomedicine and Prevention, University of Rome “Tor Vergata”, Via Montpellier 1, 00133 Rome, Italy; 2 Department of Biology, University of Rome “Tor Vergata”, Via della Ricerca Scientifica 1, 00133 Rome, Italy; 3 Neuromed, I.R.C.C.S, Pozzilli, Italy

## Abstract

The identification of new predictive biomarkers and therapeutic target for tailored therapy in breast cancer onset and progression is an interesting challenge. OLR-1 gene encodes the cell membrane receptor LOX-1 (lectin-like oxidized low-density lipoprotein receptor). We have recently identified a novel alternative OLR-1 isoform, LOX-1Δ4, whose expression and functions are still not clarified. In the present paper, we demonstrated that LOX-1 is overexpressed in 70% of human breast cancer (*n* = 47) and positively correlated to the tumor stage and grade (*p* < 0.01). Observations on LOX-1 and its splice variant Δ4 pointed out a different expression pattern correlated to breast cancer phenotypes. Overexpressing LOX-1 and LOX-1Δ4 in vitro, we obtained a strong enhancement of proliferative rate and a downregulation of cell death-related proteins. In addition, we observed a strong modulation of histone H4 acetylation and Ku70, the limiting factor of DNA double-strand breaks repair machinery implied in apoptosis inhibition and drug resistance acquisition. Moreover, LOX-1Δ4 overexpression is able to increase proliferation in a non-tumorigenic epithelial cell line, MCF12-F, acting as an oncogene. Altogether, these results suggest that LOX-1 may acts as a molecular link among metabolism, inflammation and cancer, indicating its potential role as biomarker and new molecular target, representing an attractive and concrete opportunity to improve current strategies for breast cancer tailored therapy.

## Introduction

A strong correlation between metabolic disorders, tumor development, and “multidrug resistance” acquisition has been identified, thus, targeting cancer metabolic pathways is a hot topic for drug discovery^1^. Epidemiological studies suggest an association between dysregulated metabolism and carcinogenesis^2^, supporting a link among obesity, metabolic syndrome, insulin resistance, inflammation and increased risk of cancer^1,3^. Excess of adiposity is associated with late-stage disease and poor prognosis in breast cancer^4,5^. Metabolic syndrome is characterized by elevated circulating concentrations of oxidized LDL (oxLDL) that are captured and internalized by scavenger receptors (SRs)^6^. A key role of SR-dependent oxLDL signaling in atherogenesis is well established; however, its contribution to susceptibility to cancer has not been addressed.

The recognition, internalization and degradation of oxLDL are mediated by the cell membrane receptor lectin-like oxidized low-density lipoprotein receptor (LOX-1). The OLR-1 gene (OMIM*602601), encoding LOX-1, is primarily expressed in vascular cells and vasculature-rich organs and is activated in response to oxidized LDL, angiotensin II, TNFα, and other stress stimuli^7^. In endothelial cells and macrophages, LOX-1 activation triggers inflammatory and hypoxia pathways, thinly connected with cancer insurgence and progression. Clinical trials phase II demonstrated that cholesterol-lowering atorvastatin, exerts anti-tumoral effect on breast cancer by decreasing proliferation, as measured by Ki67^8^, supporting the closed link between lipid metabolism and cancer insurgence^9^. The biological mechanisms behind the antiproliferative effects seem to be linked to the role that statins exert on LOX-1 binding. The recent publication by Biocca et al. demonstrated that atorvastatin stabilizes the dimer assembly of LOX-1 receptors in vivo and enables the receptor–substrate coupling impairing its physiological function, suggesting the involvement of LOX-1 in breast cancer^10,11^. OLR-1 gene is alternatively spliced both in mice and in humans^12,13^. In mice, we identified two splice variant forms (Δ3Δ5Olr1 and Δ2Δ5Olr1), expressed mainly during the principal developmental stages of cardiogenesis. Δ2Δ5Olr1 isoform presented a peculiar cytoplasmic diffuse dot-shaped signal that was also found in the nucleus. Moreover, this isoform activates, in vitro, inflammatory and hypoxia genes (NF-kB, Mmp9, Hif1α, VEGFα)^12^. In human, we identified another splice variant, the splice variant 2 (NM_001172632). This isoform lacks exon 4, so we called it LOX-1∆4 (also named OLR-1∆4, Rizzacasa B et al. 2017);^13^ the skipping of exon 4 causes the shifting of the reading frame causing a putative encoded protein shorter than LOX-1 with a completely different C terminus^13^. No data on the expression and functional role of LOX-1Δ4 are still available.

Recently, Hirsh and colleagues^4^ identified a common transcriptional signature involving inflammatory and metabolic pathways, which links the cancer gene signature to metabolic diseases. Interestingly, OLR-1 is a gene participating to this signature. Microarray analysis on OLR-1 KO mice, demonstrate that the abrogation of LOX-1, caused profound inhibition of rate-limiting enzymes involved in lipogenesis, including fatty-acid synthase (FASN) (5.9-fold), ATP-citrate lyase (1.7-fold), acetyl-coenzyme A carboxylase alpha (1.9-fold), stearoyl-CoA desaturase 1 (fivefold) and ELOVL family member 6, elongation of long chain fatty acids (threefold), suggesting that LOX-1 may have several pro-oncogenic actions based on activation of NF-kB signaling pathway, resulting in inhibition of apoptosis and stimulation of proliferation, and on activation of de novo lipogenesis^14^. Intriguingly these studies pointed out novel functions exerted by LOX-1 as a potent regulator of lipogenesis, regulating FASN expression, and its pro-oncogenic activity in tumors suggesting a link between obesity and susceptibility to breast cancer. Actually, previous observations on alterations of different and specific fatty-acid metabolic enzymes in the genesis and progression of breast cancer, further categorize the relevance of specific metabolic pathways to individuals intrinsic molecular subtypes of breast cancer^15,16^.

In particular, it seems that the three principal molecular phenotypes of breast cancer are characterized by different dysregulation of fatty-acid metabolism^17^. In fact, based on mRNA expression data, the less-aggressive luminal subtypes appear to rely on balance between de novo fatty-acid synthesis and oxidations as source of biomass and energy requirements, whereas basal-like receptor negative subtype overexpresses genes involved in the utilization of exogenous fatty acid and human epidermal growth factor receptor 2 (HER-2) enriched potentiates de novo synthesis^15^. The potential for targeting different metabolic pathways could help to design new molecular targets to improve a tailored therapy to individual subtypes. Here, we analyzed the expression of LOX-1 in human breast cancer tissues in relation to the classical prognostic factors strictly connected to the therapeutic treatment (estrogen receptor/progesterone receptor (ER/PR), HER-2) characterizing the role of LOX-1 and its splice variant Δ4, in vitro, in the neoplastic context of three different molecular phenotypes (MCF-7, MDA-MB-231, SK-BR-3). We found a strong modulation of LOX-1 in luminal, triple negative and HER-2-expressing tumors in respect to the healthy mammary tissues of the same individuals. Observations on LOX-1 and its splice variant Δ4 point out a different expression pattern and subcellular localization in breast cancer phenotypes. Overexpressing LOX- 1 and LOX-1Δ4 in vitro we obtained a strong enhancement of proliferative rate, a downregulation of cell cycle and cell death-related proteins. In addition, we observed a strong modulation of histone H4 acetylation and Ku70, the limiting factor of DNA double-strand breaks repair machinery implied in apoptosis inhibition and drug resistance acquisition. These data suggest a potential role of LOX-1-different isoforms in breast cancer insurgence and progression, underlying that their different role depends on the milieu of the individual breast cancer molecular subtype. In this view, LOX-1 and its splice variant could be considered functional biomarkers and potential targets for new tailored strategies, involving inhibitory drugs delivering to the patient.

## Results

### LOX-1 is overexpressed in breast cancer

**Fig. 1:**
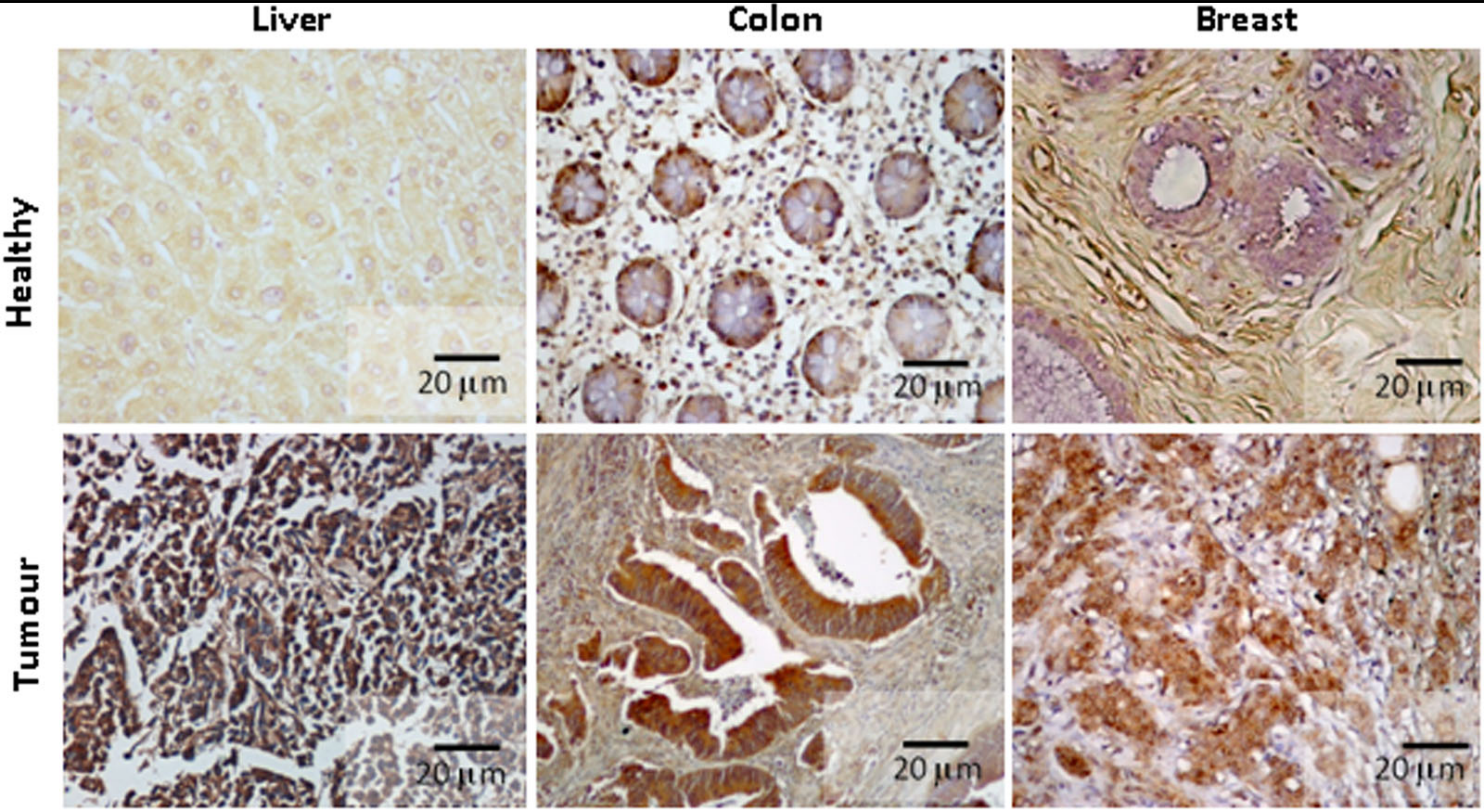
Immunohistochemical analysis of LOX-1 expression in normal and tumoral tissues. A strong increase of LOX-1 expression was observed in tumoral tissues compared with healthy counterpart aside the neoplasia of the same individuals (tumors vs healthy: *p* < 0.01)

Preliminary observations indicated a stronger LOX-1 expression in tumorigenesis. Immunohistochemical analysis of normal and tumoral tissues pointed out a strong upregulation of LOX-1 expression in tumoral tissues of liver, colon and breast as compared with healthy counterpart of the same patients (Fig. 1a). These results on protein expression are supported by data from PanCancerRNAseq database of European Bioinformatic Institute.

**Fig. 2:**
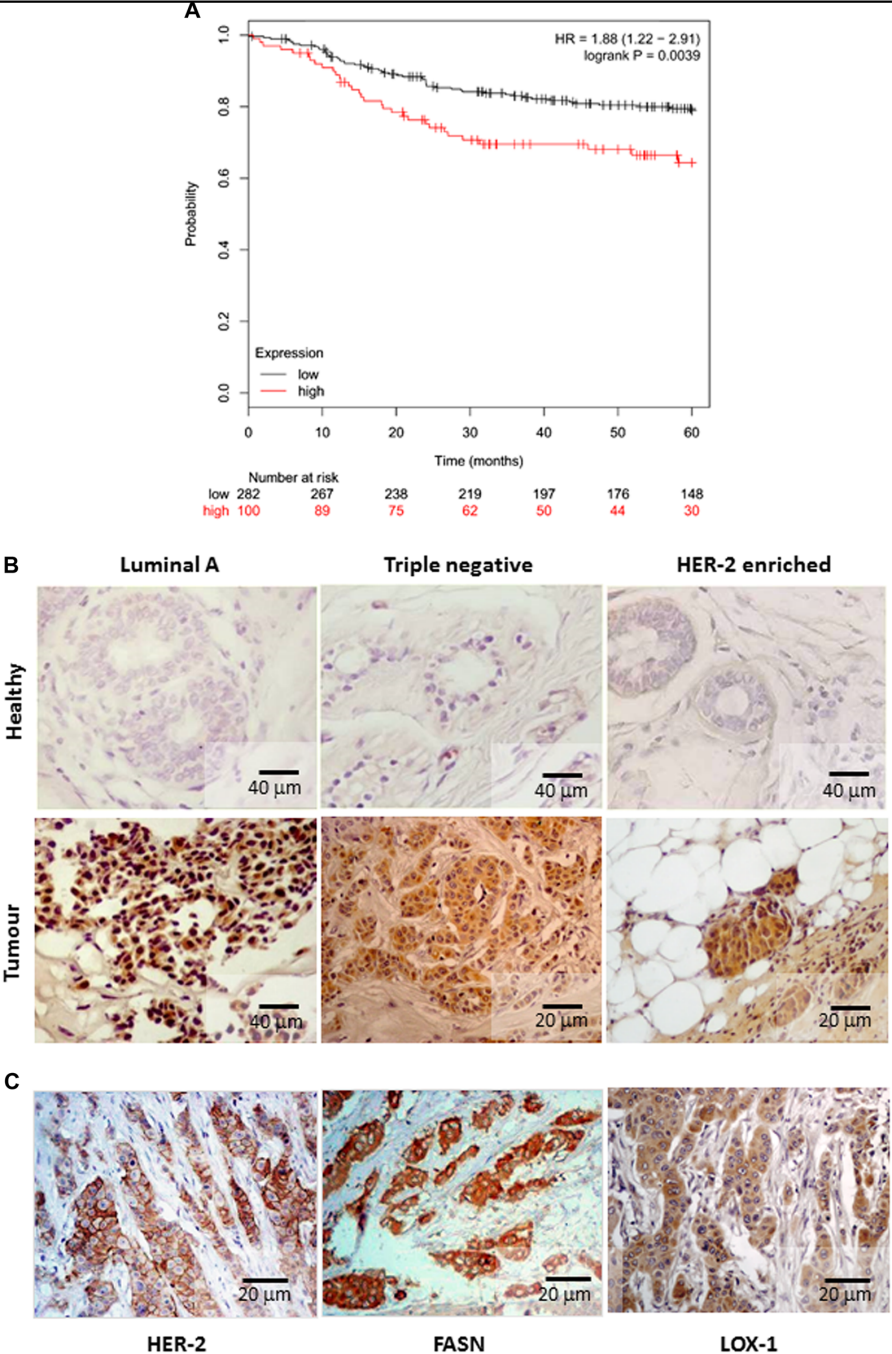
LOX-1 expression and breast cancer distant metastasis free survival (DMFS). **a** Kaplan–Meier plot analysis of LOX-1 expression level (low or high) on breast cancer distant metastasis free survival (DMFS). **b** Immunohistochemical analysis of LOX- 1 expression in breast cancer phenotypes and healthy counterpart of the same individuals. **c** Correlation among HER-2 amplification, FASN and LOX-1 overexpression in HER-2-enriched phenotypes (HER-2- FASN positive vs LOX-1: *p* < 0.01)

We focused the study on breast carcinomas characterized by grading and staging according to WHO and TNM 2009 classifications and grouped according to St. Gallen consensus 2013. In particular, we observed in ~70% (32 out of 47) of breast cancer tissues, a strong upregulation of LOX-1 (tumors vs controls: *p* < 0.01), positively correlated to the tumor stage and grade (Table 1). In fact, we observed a strong LOX-1 overexpression in invasive and metastatic breast cancer tissues as compared with the normal tissues of the same individuals aside the neoplasia (*p* < 0.01). The role of LOX-1 in breast cancer survival was further confirmed by analyzing the association of LOX-1 expression in breast cancer tissues to patient’s outcome in public datasets. Kaplan–Meier survival plot reveal that patients with a high LOX-1 expression in tumor tissues had significantly shorter cancer-specific distant metastasis-free survival compared  to those with a low LOX-1 expression (*p* = 0.0039), confirming the relevance of LOX-1 expression in breast cancer progression and survival (Fig. 2a). Interestingly, as show in Table 2, LOX-1 expression and localization in subcellular compartment were variable in tumoral tissues and related to prognostic factors expression (ER, PR and HER-2). Immunohistochemistry showed a peculiar nuclear LOX-1 localization in tumors ER^+^/PR^+^ (Luminal A). In triple-negative tumors, LOX-1 expression was mainly observed in the cytoplasm, weakly in the nucleus, and in some cases secreted in the cellular stroma, whereas in infiltrating HER-2-enriched tumors, LOX-1 was found strongly expressed in the cytoplasm and in many cases in the nuclei (Fig. 2b; Table 2).

**Table 1:** LOX- 1 protein expression, detected by immunohistochemistry, in human breast cancer tissues

LOX-1 expression in invasive breast cancer		
	G_1_-G_2;_ any T; N_(0)_ 30% (*n* = 15)	G_2_-G_3;_ any T; N_(1)_/N_(2)_; M_(0)_/M_(1)_ 70% (*n* = 32)
Weak, % (*n*)	7 (1)	0 (0)
Moderate, % (*n*)	73 (11)	12.5 (4)
Strong, % (*n*)	20 (3)	87.5 (28)

Number of cases was shown as percentage. In parentheses, the absolute numbers were reported

**Table 2:** Breast cancer cases analyzed and classified according to St. Gallen consensus 2013

Breast cancer phenotype	Main clinical features				Cases % (*n* = 47)	Expression of			
	ER	PR	HER-2 (score)	Ki67		*LOX-1*		*FASN*	LOX-1 preeminent isoforms
						Cytoplasm	Nucleus		
Luminal A	> 60%	> 20%	≤1+	<14%	60%	+	++	40%	LOX-1Δ4
HER-2 enriched	−	−	≥2+	NA	20%	+++	+	90%	LOX-1
Triple negative	−	−	≤1+	NA	20%	++	+	40%	LOX-1

*ER* estrogens receptor, *PR* progesterone receptor, *HER-2* human epidermal growth factor receptor 2, *NA* not applicable

The signal intensity depends on the amount of protein expressed and results to be stronger in HER-2-enriched tumors as compared with triple negative (*p* < 0.01) and luminal tumors (*p* < 0.01). In order to explain the strong LOX-1 expression observed in HER-2 enriched FASN-positive tumors, FAS expression was evaluated by immunohistochemistry on serial sections of the same tissue.

### Fatty-acid synthase expression in human tumours is correlated to LOX-1

FAS protein was found significantly increased in the cytoplasm of tumors analyzed (tumors vs controls: *p* = 0.003). Only 20% of tumors displayed absence or weak FAS staining in the cytoplasm of neoplastic cells. In particular, 91% of breast carcinomas HER-2-enriched and 40% of ER-positive breast cancer displayed positive staining (1 + , 2 + ), as well as 40% of triple negative. A strong LOX-1 overexpression was observed in HER-2-enriched FASN-positive tumors, suggesting the correlation among HER-2 amplification, FASN and LOX-1 overexpression in this phenotype (HER-2-FASN positive vs LOX-1: *p* < 0.01) (Fig. 2c). Moreover, LOX-1 was found expressed with a different intensity and localization also in Luminal A (ER^+^/PR^+^) and triple negative FASN-expressing tumor (Table 2).

### LOX-1 isoforms are differently expressed in human cancer tissues from different breast cancer phenotypes

**Fig. 3:**
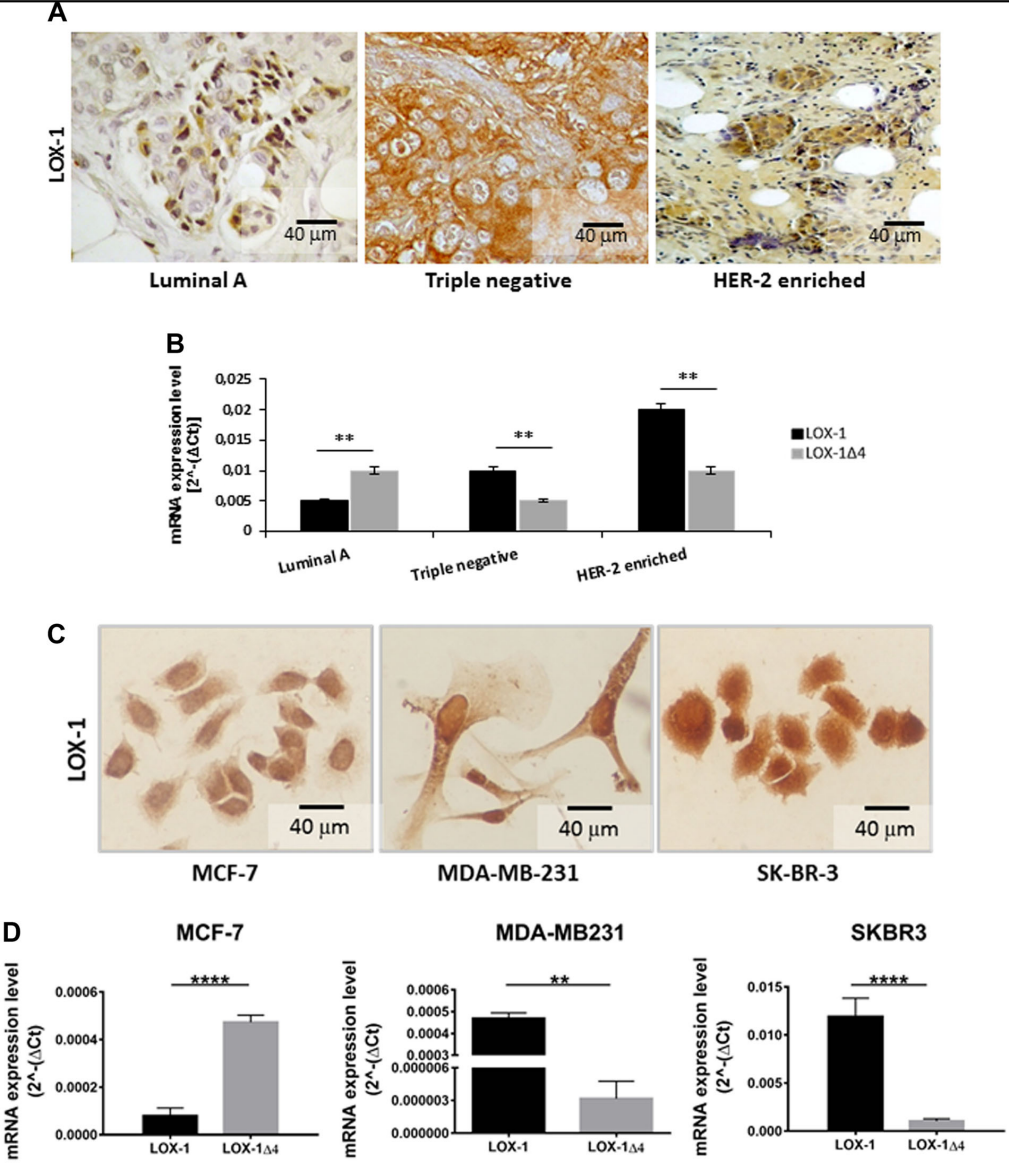
LOX-1 expression pattern in breast cancer cells and tissues. Immunostaining **a–c** and RNA **b–d** analysis of LOX-1 isoforms pattern expression in different breast tissues **a**, **b** (**p < 0.005) and cell lines phenotypes **c**, **d** (**p < 0.005; ****p < 0.00005). qRT-PCR analysis shows that LOX-1 and its LOX-1Δ4 isoform are both expressed in breast cancer cells analyzed but at different levels depending on the cell line phenotype. Values are mean ± SD, and *n* = 3

As immunohistochemistry was not able to discriminate LOX-1 and LOX-1Δ4 proteins, we analyzed mRNA expression obtained from nine breast cancer samples (three out of nine luminal A, three triple negative, three HER-2 enriched) of same individuals previously characterized by immunohistochemistry, as shown in Fig. 3a. RT-PCR was performed using two different set of primers, as described in materials and methods. We observed that LOX-1 and its splice variant LOX-1Δ4 were different expressed in breast cancer phenotypes. As reported in Fig. 3b, Luminal A phenotype displayed a strong upregulation of LOX-1Δ4 splice variant. On the contrary, in triple negative and HER-2-enriched tissues, full-length LOX-1 isoform was the preeminent form, indicating a different expression pattern in the breast cancer phenotypes. HER-2-enriched FASN + breast tumors expressed LOX-1 four times more than luminal A tumours, and two times more than basal-like tumors.

### LOX-1 mRNA expression pattern in breast cancer cell lines correlates to breast cancer tissues phenotypes

In order to better define LOX-1 and its splice variant Δ4 in breast cancer, we analyzed their expression in three different phenotypes of breast cancer cell lines, MCF-7 (ER^+^/PR^+^, luminal A), MDA-MB-231, deriving from a triple-negative tumor and SK-BR-3 (HER-2 enriched). The immunocytochemical analysis for LOX-1 expression, performed 48 h after seeding as described in Materials and methods, highlighted a strong expression and different subcellular localization of LOX-1 among the three different cell lines. LOX-1 in MCF-7 displayed a nuclear localization, in MDA-MB-231 and SK-BR-3 cell both nuclear and cytoplasmatic (Fig. 3c). In SK-BR-3, LOX-1 displayed the higher expression, confirming results observed in human HER-2-enriched breast cancer tissue. Hence, we analyzed the expression of LOX-1 and its splice-form mRNA, by quantitative real-time PCR (qRT-PCR). Data demonstrated a different expression pattern of LOX-1 splice isoforms depending on the breast cancer phenotype as previously observed in human breast cancer tissues. As reported in Fig. 3d, in MCF-7 ER^+^/PR^+^, LOX-1Δ4 was the prevalent expressed isoform, in MDA-MB-231 and SK-BR-3 LOX-1 results overexpressed as compared with LOX-1∆4. It is worth of note that SK-BR-3 breast cancer cells displayed an evident higher amount of LOX-1 as compared with the other phenotypes observed. These data confirm results observed in human cancer tissues. To further analyze the functional role of LOX-1 and LOX-1Δ4 isoform, we overexpressed them in cancer cells.

### LOX-1 strongly affects cell proliferation rate

**Fig. 4:**
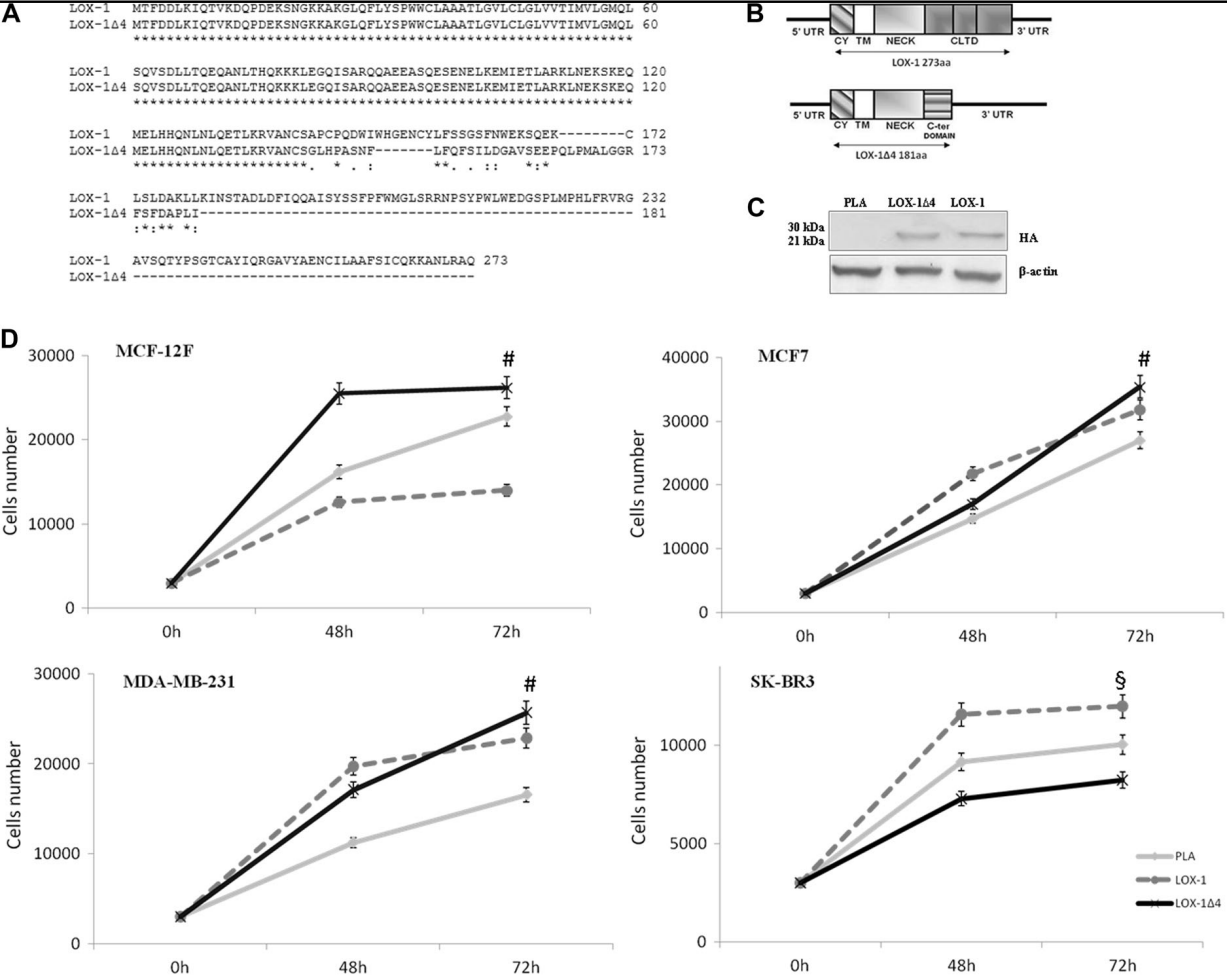
Effect of LOX-1 isoforms overexpression in breast cancer cell lines. **a** Alignment and comparison of aminoacidic sequences of LOX-1 full-length form (NP_002534.1) and LOX-1Δ4 (NP_001166103.1). **b** Schematic representation of protein products of LOX- 1 and LOX-1Δ4. 5’ UTR, 5’ untranslated region; CY, cytoplasmic domain; TM, transmembrane domain; NECK, neck domain; CLTD, C-terminal lectin type domain; 3’ UTR, 3’ untranslated region. **c** Western blot analysis of HA-tagged LOX-1 isoforms transfected into MDA-MB-231 cells. **d** Effects of the overexpression of LOX-1 and LOX-1Δ4 isoforms in breast cancer cell lines. Empty plasmid was used as control (PLA). Values are mean ± SD, and *n* = 3 (^#^*p* < 0.01 PLA vs LOX-1Δ4; ^§^*p* < 0.05 PLA vs LOX-1)

LOX-1 and LOX-1Δ4 splice form were amplified from SK-BR-3 mRNA, with specific primers as described in Materials and methods and subsequently cloned in pcDNA3-HA vector. Before transfection experiments, each vector insert was fully sequenced. Comparison of aminoacidic sequences and schematic representation of protein products of LOX-1 and LOX-1Δ4 are shown respective in Fig. 4a, b.

MCF-7, MDA-MB-231 and SK-BR-3 cell lines were used to characterize the effect of LOX-1 different forms overexpression on proliferative rate in the different phenotypes and MCF-12F isolated from normal mammary gland were used as control. The transfection efficiency and expression was valuated by hemagglutinin (HA) immunoblot (Fig. 4c).

As shown in Fig. 4d, in MCF-12F cell line, the overexpression of LOX-1 failed to positively modulate the proliferative rate in this cell line (PLA vs LOX-1: *p* < 0.01). On the contrary, the proliferative increase resulting from LOX-1Δ4 overexpression was evident (PLA vs Δ4: *p* < 0, 01), suggesting the Δ4 capacity to transform normal cell, acting as a new metabolic oncogene.

In MCF-7 cancer cells, LOX-1 construct is able to induce an evident proliferative increase as compared with transfected cell with empty plasmid (Fig. 4d). Moreover, also the overexpression of LOX-1Δ4 induces a proliferative advantage in MCF-7 cells as compared with cells transfected with empty plasmid, particularly evident at 72 h after transfection (PLA vs Δ4: *p* < 0.01).

In MDA-MB-231 triple-negative cancer cell line, the overexpression of LOX-1 induces an increase of proliferation as compared with cell transfected with empty vector (PLA vs LOX-1: *p* < 0.01).

LOX-1Δ4 overexpression induces an increase of proliferation as compared with control, and after 72 h confers a higher proliferative advantage, as compared with cell transfected with empty vector (PLA vs Δ4: *p* < 0.01) and cell transfected with LOX-1 (LOX-1 vs Δ4: *p* < 0.05) (Fig. 4d).

Peculiar effect of LOX-1 and its isoform Δ4 on cell proliferation rate was observed in SK-BR-3 cells. In this cell line, where HER-2 is amplified and FASN overexpressed, the proliferation increase is evident only in LOX-1-transfected cells as compared with control (PLA vs LOX-1: *p* < 0.05). On the contrary, the overexpression of Δ4 spice variant seems to decrease the proliferation rate (Fig. 4d).

### LOX-1Δ4 affects Ku70 and histone H4 acetylation pattern in MCF-7 cell line

**Fig. 5:**
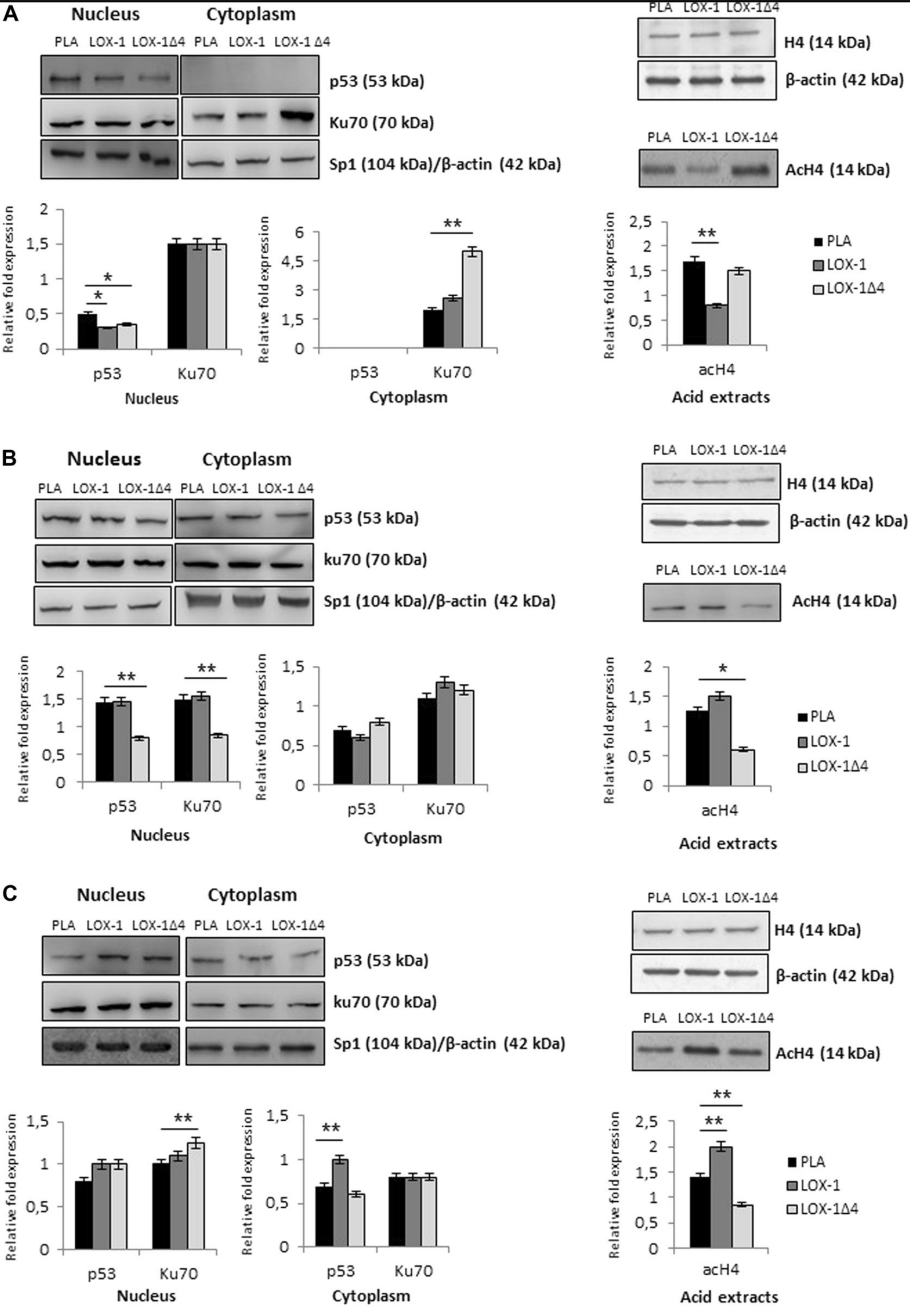
LOX-1 splice variants affects p53, Ku70, and histone H4 acetylation pattern in different breast cancer cell lines. **a** A downregulation of p53 expression was observed in all transfected cells. In LOX-1Δ4-transfected cells we observed a strong increase of Ku70 expression in the cytoplasm. A significant reduction of histone H4 acetylation was observed in LOX-1-transfected cells. **b** A decrease of p53 level in the nucleus is evident in LOX-1Δ4-transfected cells and a decrease of Ku70 level was observed in the nucleus in LOX-1Δ4transfected cells. A strong decrease in histone H4 acetylation in MDA-MB-231 LOX-1Δ4transfected cells was detected. **c** In the cytoplasm we observed a light p53 augmentation in LOX-1-transfected cells. Ku70 increases in the nucleus. The overexpression of LOX-1 seems to increase histone H4 acetylation, on the contrary LOX-1Δ4 induce a decrease of acetylation pattern. In all cell lines analyzed, histone H4 was used as internal control. Student’s *t* tests were used to verify statistically significant (*p < 0.05; **p < 0.01)

Nuclear and cytoplasmic extracts were prepared from transfected cells. As shown in Fig. 5a and summarized in Table 3, we observed a downregulation of p53 expression in cells transfected with LOX-1 and LOX-1Δ4 (PLA vs LOX-1: *p* < 0.05; PLA vs LOX-1Δ4: *p* < 0.05). MCF-7 cell line displays p53 wild type, therefore its downregulation suggests an attempt to block p21 transcription and its proapoptotic action. Ku70, protein involved in DNA repair in the nucleus and apoptosis inhibition in the cytoplasm, seems not to be modulated in the nucleus in all transfected cells. On the contrary in LOX-1Δ4-transfected cells we observed a strong increase of Ku70 in the cytoplasm (PLA vs LOX-1Δ4: *p* < 0.01) where it exerts a strong antiapoptotic action-binding BAX. These data confirm a strong inhibition of apoptosis induction affected by LOX-1Δ4 splice variant. Moreover, in order to define the role of the different LOX-1 splice variants in epigenetic regulation, protein acid extraction was performed from cell lines transfected with LOX-1 forms. As shown in Fig. 5a, in MCF-7 cell line, we observed a reduction of histone H4 acetylation in cells transfected with LOX-1 and LOX-1Δ4 splice variant, more evident in cells transfected with LOX-1 construct as compared with cells transfected only with empty vector (PLA vs LOX-1: *p* < 0.01).

**Table 3:** Schematic report of relevant effect of LOX-1 isoforms on proliferation, p53, Ku70, and Ac.H4 modulations in MCF-7, MDA-MB-231 and SK-BR-3 cell lines (N.D: non detectable; =: no modulation observed)

			*P53*		*KU7O*		*Ac.H4*
		Proliferative advantage	Nucleus	Cytoplasm	Nucleus	Cytoplasm	Nucleus
MCF-7	*LOX-1*	↑	−	N.D.	=	+	—
	*LOX-1Δ4*	↑↑	−	N.D.	=	++	−
MDA-MB-231	*LOX-1*	↑	=	=	=	+	+
	*LOX-1Δ4*	↑↑	—	+	—	=	—
SK-BR-3	*LOX-1*	↑↑	+	+	=	=	++
	*LOX-1Δ4*	↓↓	+	=	+	=	—

### Ku70 and p53 protein expression and histone H4 acetylation are modulated in MDA-MB-231 in LOX-1Δ4-transfected cells

In MDA-MB-231-transfected cells, we observed the expression level of the same gatekeeper and caretaker proteins. In this cell line, p53 is inactivated by a point mutation [(TP53): c.524 G > T] and as shown by Hui and colleagues^18^, p53mut stabilized by elevated phospholipase D activity, can contribute to the suppression of apoptosis. The inactive cytoplasmic p53 was found in all the experimental groups. A light increase of p53 level in the cytoplasm correlates with a decrease of nuclear p53 (PLA vs LOX-1Δ4: *p* < 0.01), suggesting a possible translocation from the nucleus induced by LOX-1Δ4.

On the contrary a strong decrease of Ku70 repair protein in the nucleus was noted in cells transfected with LOX-1Δ4 (PLA vs LOX-1Δ4: *p* < 0.01), concomitantly a light increase of Ku70 in the cytoplasm was observed in LOX-1 and LOX-1Δ4-transfected cells, suggesting an increased apoptotic inhibition. Moreover, we observed a strong decrease in histone H4 acetylation in MDA-MB-231 in LOX-1Δ4-transfected cells (PLA vs LOX-1Δ4: *p* < 0.01), the splice variant that confers the higher proliferative advantage in this cell line compared with control (Fig. 5b; Table 3).

### LOX-1 induces an hyperacetylation of histone H4 in SK-BR-3-transfected cells

In HER-2 amplified breast cancer cell line (SK-BR-3), p53 mutation [(TP53): c.524 G > A] displayed an attenuated tumor suppressor activity in the regulation of transcription, colony formation and apoptosis^19^. The analysis of protein extracts showed a p53 augmentation in the cytoplasm of LOX-1-transfected cells (PLA vs LOX-1: *p* < 0.01). No significant modulation was evident in cells transfected with LOX-1Δ4. Moreover, a mild Ku70 increase was present in the nucleus in transfected cells with LOX-1Δ4 (PLA vs LOX-1Δ4: *p* < 0.01), suggesting a potentiating effect in DNA repair mechanism, whereas no evident modulation of Ku70 expression was noted in the cytoplasm. In SK-BR-3-transfected cells, the overexpression of LOX-1 seems to increase histone H4 acetylation (PLA vs LOX-1: *p* < 0.01), on the contrary LOX-1Δ4 induces a decrease of acetylation pattern (PLA vs LOX-1Δ4: *p* < 0.01) (Fig. 5c; Table 3).

## Discussion

Recently, cancer research has focused on dysregulated metabolism in cancer cells and metabolic reprogramming is now considered a hallmark of cancer, therefore targeting cellular metabolism is a promising strategy to overcome drug resistance in cancer therapy. The present work focuses on the role of LOX-1 in breast cancer, evidencing for the first time the different expression of LOX-1 and its splice variant Δ4 in different human breast cancer molecular phenotypes. We found a strong upregulation of LOX-1 in breast cancer tissues, whose expression and localization were related to the specific phenotypes. We observed a peculiar nuclear localization in luminal phenotypes, a cytoplasmic expression of LOX-1 in triple-negative tumors and the presence of a high amount of the protein as in the cytoplasm as in the nuclei in HER-2-enriched subtype, suggesting the existence of different pathways and compartmental distribution of LOX-1 in each phenotype. Hence, the characterization of LOX-1 and its splice variants expression on different breast cancer cell lines points out a different effect in each subtype analyzed; this could depend on the specific metabolic milieu in the different breast cancer phenotypes. Data from tissues observations underline the peculiar overexpression of LOX-1Δ4 in luminal phenotype, on the contrary the full-length LOX-1 isoform was predominantly expressed in the other two molecular profiles. The expression of LOX-1 and its splice variants pattern observed in vitro, confirms the observation on cancer tissues, and underlines that MCF-7 luminal breast cancer-derived cell line, is the only one where LOX-1Δ4 is overexpressed, confirming the pattern observed in luminal breast cancer tissues. The expression and the function of this form in human tissues are still obscure, but we demonstrated that its overexpression is the only one that is able to increase proliferation in MCF-12F cell line, indicating the oncogenic potential of this protein.

On the contrary in triple-negative and HER-2-enriched tumors full-length LOX-1isoform was the preeminent expressed form. Transfection experiments indicate that the action of LOX-1 and its splice variant is specific and related to individual molecular phenotypes probably depending to metabolic pathways activated. In particular, in HER-2-enriched breast cancer, we noticed a strong LOX-1 overexpression as compared with other phenotypes, suggesting a possible involvement of FASN in modulating proliferative rate of this breast cancer cells suggesting a potential connection of LOX-1 in FASN-HER-2 axis. Results obtained on epigenetic modulation point out that the effect on acetylation pattern is specific for each splice variant and depends on the breast cancer molecular phenotype and its intracellular context. These results could be connected to the metabolic state of the different breast cancer molecular subtypes^20^. The overexpression of LOX-1 and its splice variant Δ4 affects p53 expression and localization, indicating a potential connection between metabolic factors and regulation of cell cycle and proliferation.

Furthermore, we demonstrated that LOX-1 could affect the DNA repair protein Ku70 and its antiapoptotic action. In fact, we found a strong modulation of Ku70 expression in the cytoplasm of MCF-7 and MD-MB-231 where, as we previously published in tumors^21^, it binds BAX inhibiting its activation and translocation into mitochondria. Concomitantly in MDA-MB-231 LOX-1Δ4-transfected cell line is also evident a strong decrease of Ku70 potential DNA-repairing action in the nucleus. On the contrary in SK-BR-3, Ku70 results upregulated in the nucleus where it could have a principal role in DNA repair activity conferring a potential resistance from DNA damage of chemo and radiotherapy, modulating the DNA repair efficiency. LOX-1 and its splice variant Δ4 modulate the epigenetic state of cancer cells as we have previously shown^22^. In MCF-7, the overexpression of the different forms induces a reduction of histone acetylation, usually recurred in high-grade breast cancer, particularly evident in LOX-1-transfected cells. Conversely, in MDA-MB-231 and SK-BR-3, LOX-1 induces a light increase in H4 acetylation, whereas LOX-1Δ4 induces a strong decrease of acetylation pattern. The present study suggests that LOX-1 and its splice variant Δ4 could exert an oncogenic role on breast cancer tissues, and the expression pattern could be specifically modulated in different breast cancer phenotypes, concurring to affect the proliferation rate, the apoptosis induction, the DNA repair processes, and the epigenetic state of cancer cells. Moreover, data from Kaplan–Meier plot point out that LOX-1 overexpression in breast cancer tissues correlates to a shorter cancer-specific distant metastasis-free survival, suggesting its involvement in metastasis formation and chemoresistance acquisition.

Completely understanding LOX-1 and its splice variant Δ4 molecular pathways in breast cancer molecular phenotypes could represent a new challenge for targeting tumor metabolic pathway involved in proliferation, drug resistance acquisition and to improve a tailored therapy to individual subtypes.

## Materials and methods

### Patients’ characteristics and tissue samples

We retrospectively analyzed 47 patients affected by mammary carcinomas presented at Policlinico Tor Vergata between November 2014 to November 2017. Formalin-fixed, paraffin-embedded surgical findings stored at −80 °C were analyzed. The histological samples were classified by means of haematoxylin and eosin staining according to the TNM 2009 classifications. Table 2 shows the main clinical features of human mammary carcinomas analyzed grouped according to St. Gallen consensus 2013. The ethics committee of “Policlinico Tor Vergata” (approval reference number # 143/14) approved all experiments described in the present study. All experimental procedures were carried out according to The Code of Ethics of the World Medical Association (Declaration of Helsinki). Informed consent was obtained from all patients prior to surgery. Specimens were handled and carried out in accordance with the approved guidelines.

### Analysis of public breast cancer data sets

The Kaplan–Meier plotter is capable of assessing the effect of 54,675 genes on survival using 10,293 cancer samples based on a meta-analysis of biomarkers. Kaplan–Meier plot, the hazard ratio with 95% confidence intervals and log rank *P* value were calculated. This platform can be reached over the internet via http://www.kmplot.com/breast cancer.

### Immunohistochemistry

Sections, 5 µm thick, were deparaffinized and rehydrated through xylene and alcohol. Endogenous peroxidases were blocked in methanol solution with 3% hydrogen peroxide for 20 min. After three washes with a solution of filtered TBS1X/0.05% Triton X-100, sections were incubated with serum for 1 h at room temperature and then with the primary antibody diluted in a solution of TBS1X/2% bovine serum albuin (BSA) for 60 min. Subsequently, the sections were incubated for 45 min at room temperature with the secondary antibody properly diluted in TBS1X/20% BSA and then they were incubated for 30 min at room temperature with streptavidin (ImmunoCruzStaining System sc-2053, Santa Cruz Biotechnology, Inc.). The staining was completed after a short incubation with a freshly prepared substrate—chromogen, diaminobenzidine (DakoCytomation). Sections were washed extensively in water and nuclei were counterstained with hematoxylin followed by dehydration with increasing concentration of ethanol. The procedure was performed for detection of LOX-1 (anti-LOX-1 ab60178 Abcam, rabbit polyclonal antibody) and FASN (anti-FASN K-20 sc-16146 Santa Cruz Biotechnology, Inc. goat polyclonal antibody). All cases were digitally scanned by iScan (Produced by BioImagene, Now Roche-Ventana) with Scanning Resolution 0.46 µm/pixel at × 20 with Scanning Resolution 0.46 µm/pixel at × 20. The IHC signals of LOX-1 were measured using an automated image analyzing system (MECES). The intensity of LOX-1staining was scored as negative/weak (0), moderate (1), strong (2). The cell positivity was also scored as < 10 % (0), from 10% to 25% (1) from 26% to > 50% (2). For evaluation of results, *t* test was performed.

### Cells lines, cultures, and transfection

MCF-12F non cancerous mammary cells (CRL-10783, ATCC), MCF-7 breast cancer cells (HTB-22, ATCC), MDA-MB-231 (HBT26, ATCC), SK-BR-3 (HBT30, ATCC) purchased from American Type Culture Collection were grown in complete culture medium, according to condition ATCC suggested. LOX-1 isoforms (OLR-1, OLR-1Δ4) were cloned into pcDNA-HA. Transient transfections were performed, by using Calcium Phosphate Transfection Kit (Invitrogen). After an overnight culture, cells were transfected with empty plasmid vector (as control) and LOX-1 isoforms plasmid vectors. Complete growth medium was added to cultures after following 24 h.

### Evaluation of cells growth

Cells were seeded in 96-multiwell plates at 15 × 10^3^ cell/cm^2^ and were transfected with LOX-1 and OLR-1Δ4 isoform. Cell count was performed after 48 and 72 h post transfection and the number of attached cells was determined using Burker counting chamber, by Trypan Blue method. MTT assay was performed in the same samples. All experiments were performed in triplicate. Transfected cells with empty plasmid were used as controls. Transfection efficiency was validated by qRT-PCR.

### Immunocytochemistry

MCF-7, MDA-MB-231 and SK-BR-3 cell lines were plated in Lab Tek II Chamber Slides (Nalge Nun Int.) at 25 × 10^3^ cells/cm^2^ concentration. After transfection, the medium was removed and cells were fixed in formalin 10% solution (Sigma/Aldrich) for 5 min and stored at 4 °C in 1 × PBS. Cells were permeabilized with 0.5 % Triton X-100 and 0.05 % Tween-20 (Bio-Rad Laboratories, Munchen) in PBS. Subsequently, cells were washed two times with 1 × PBS and non-specific sites were blocked with serum incubation. Immunocytochemistry analysis was performed for LOX-1 detection as described in Immunohistochemistry section.

### Western blot

After medium withdrawal, detachment with trypsin-ethylenediaminetetraacetic acid (EDTA) and washing in PBS, cell pellets were incubated at − 80 °C overnight. Solution A (HEPES-KOH 10 mm, 1.5 mm MgCl_2_, 10 mm KCl, EDTA 1 mm, 1 mm PMSF, 1 mM DTT, 20 mm NaF, 1 mm Na_4_P_2_O_7_) was added to cell pellets. Cells were allowed to swell on ice for 30 min and were resuspended with addition of Nonidet P-40 10%. After 10 min, cytoplasmic membranes were ruptured through insulin syringes. Acid-soluble proteins were extracted according to the protocol for histone proteins (UpState). Nuclear and cytoplasmic fractions were separated by centrifugation in a microfuge at 12,000 r.p.m. Solution B (HEPES-KOH 20 mm, 1.5 mm MgCl_2_, 420 mm NaCl, EDTA 1 mm, 2% Glycerol, 1 mm PMSF, 1 mm DTT, 20 mm NaF, 1 mm Na_4_P_2_O_7_) was added to nuclear pellet that was incubated 30 min on ice. Nuclear proteins were separated by centrifugation in a microfuge at 12,000 r.p.m. Protein content in nuclear and cytoplasmic extracts was determined in triplicate by Bradford assay (Bio-Rad Protein Assay, Bio-Rad Laboratories, Munchen). Nuclear and cytoplasmic proteins extracted as above (20 μg) were denatured under reducing conditions (1.0% β-mercaptoethanol) in sample buffer and separated by 10% SDS-PAGE. Proteins were transferred to a polyvinylidene fluoride membrane (Hybond P, Amersham GE Healthcare) using an electro-blotting apparatus. Membranes were stained with Ponceau S dye, to check equal loading and homogeneous transfer and incubated for 1 h at room temperature with 3% skim milk (Difco Lab., MI, USA) and 0.25% Tween-20.

#### Primary antibodies

Anti-p53 (DO-1) (sc-126, Santa Cruz Biotechnology), anti-Ku70 (A-9) (sc-5309, Santa Cruz Biotechnology), anti-HA (12CA5) (sc: 57592, Santa Cruz Biotechnology), anti-acetyl-H4 (Upstate) antibodies. Filters were reprobed with anti-β-actin mouse monoclonal antibody (Sigma-Aldrich, 63103 USA) or anti-Sp1 mouse monoclonal antibody (Santa Cruz Biotechnology) to normalize respectively cytoplasmic or nuclear protein levels. Anti-H4 (F-9) (sc: 25260, Santa Cruz Biotechnology) mouse monoclonal antibody was used as internal control for acetylated H4. Filters were developed using an enhanced chemiluminescence system (Bio-Rad Clarity Western ECL Blotting Substrates).

### RNA extraction and RT-PCR

Total cellular RNA was extracted by Tri Reagent (Ambion, Inc.) according to the manufacturer’s instruction. RNA quantification was performed using spectrophotometry. In total, 1 µg of RNA was used for reverse transcription using SuperScript III (Invitrogen) and Platinum Taq DAN Polimerase (Invitrogen) for complementary DNA (cDNA) synthesis. cDNA was amplified with specific primer for LOX-1 (NM_002543) and LOX-1Δ4 (NM_001172632). Amplification protocol was performed in a thermocycler (Applied Biosystems) with following condition: LOX-1 and LOX-1Δ4 with 40 cycles at 62 °C. Ethidium-bromide-stained agarose gel was run at 100 V and the gel was acquired by scanning system. To normalize template input, β2-microglobulin transcript was amplified for each sample.

### Quantitative real time PCR

Total RNA from transfected cells was treated with DNAse (2 U/μl; Ambion) and retrotranscripted by using “High Capacity cDNA Reverse Transcription Kit” (Applied Biosystems, Waltham, MA, USA). A qRT-PCR (SYBR Green assay Applied Biosystems) assay was performed with different primers pairs specific for each LOX-1 isoforms:

LOX-1 (F:5′-GCACAGCTGATCTGGACTTCAT-3′, R:5′CCCCATCCAGAATGGAAAACT-3′);    LOX-1Δ4 (F:5′-TTGTTCAGGACTTCATCCAGC-3′, R:5′-TCGGACTCTAAATAAGTGGGG-3′);

β2-microglobulin gene was used for data normalization.

### Statistical analysis

All values provided in the text and figures are means of three independent experiments ± standard deviations (SD). Unpaired *t* -tests were used for normally distributed continuous variables to assess statistical differences (*p* ≤ 0.05 was significant) and to compare normal LOX-1 immunohistochemistry to tumoral tissues.
